# Parental and household smoking and the increased risk of bronchitis, bronchiolitis and other lower respiratory infections in infancy: systematic review and meta-analysis

**DOI:** 10.1186/1465-9921-12-5

**Published:** 2011-01-10

**Authors:** Laura L Jones, Ahmed Hashim, Tricia McKeever, Derek G Cook, John Britton, Jo Leonardi-Bee

**Affiliations:** 1 UK Centre for Tobacco Control Studies, Division of Epidemiology and Public Health, University of Nottingham, Clinical Sciences Building, Nottingham City Hospital, Nottingham, NG5 1PB, UK; 2 Division of Community Health Sciences, St George's University of London, Cranmer Terrace, London, SW17 ORE, UK

## Abstract

**Background:**

Passive smoke exposure increases the risk of lower respiratory infection (LRI) in infants, but the extensive literature on this association has not been systematically reviewed for nearly ten years. The aim of this paper is to provide an updated systematic review and meta-analysis of studies of the association between passive smoking and LRI, and with diagnostic subcategories including bronchiolitis, in infants aged two years and under.

**Methods:**

We searched MEDLINE and EMBASE (to November 2010), reference lists from publications and abstracts from major conference proceedings to identify all relevant publications. Random effect pooled odds ratios (OR) with 95% confidence intervals (CI) were estimated.

**Results:**

We identified 60 studies suitable for inclusion in the meta-analysis. Smoking by either parent or other household members significantly increased the risk of LRI; odds ratios (OR) were 1.22 (95% CI 1.10 to 1.35) for paternal smoking, 1.62 (95% CI 1.38 to 1.89) if both parents smoked, and 1.54 (95% CI 1.40 to 1.69) for any household member smoking. Pre-natal maternal smoking (OR 1.24, 95% CI 1.11 to 1.38) had a weaker effect than post-natal smoking (OR 1.58, 95% CI 1.45 to 1.73). The strongest effect was on bronchiolitis, where the risk of any household smoking was increased by an OR of 2.51 (95% CI 1.96 to 3.21).

**Conclusions:**

Passive smoking in the family home is a major influence on the risk of LRI in infants, and especially on bronchiolitis. Risk is particularly strong in relation to post-natal maternal smoking. Strategies to prevent passive smoke exposure in young children are an urgent public and child health priority.

## Background

The 2006 US Surgeon General's report on the effects of involuntary exposure to tobacco smoke concluded that passive smoking was a cause of a range of diseases of children, including acute lower respiratory infection (LRI) [1]. Those conclusions were based in part on the results of a series of systematic reviews and meta-analyses first commissioned for a report by the UK Government Scientific Committee on Tobacco and Health (SCOTH) [2], which were then updated for the Surgeon General report. The original meta-analysis of effects on LRI was published by Strachan and Cook in 1997 [3] and included papers published to 1996; the update for the Surgeon General, as well as an updated SCOTH report published in 2004 [4], included papers published to 2001.

Since 2001, many more studies of this association have been published but have not as yet been subject to meta-analysis. We have therefore updated the original Strachan and Cook review and meta-analyses of the epidemiological data to provide contemporary estimates of the effect of passive smoking on LRI in infants in the first two years of life, and to use the larger evidence base to explore the effects of pre-natal and post-natal exposure, effects of smoking by either parent, both parents or by any household member, and the effects of passive smoking on subcategories of the LRI diagnostic group. The work was carried out as part of a more extensive review of the effects of passive smoking in children, for the Royal College of Physicians [5].

## Methods

### Systematic review methods

The search strategy employed in the original Strachan and Cook systematic review and meta-analysis [3] was repeated in the current study and included a comprehensive literature search of MEDLINE (1997 to November 2010) and EMBASE (1997 to November 2010), published reviews, reference lists from identified publications and abstracts from major conference proceedings (European Respiratory Society and American Thoracic Society). No restrictions on language were imposed during the searches, but in keeping with the original strategy we report only results from papers written and published in English [3]. Studies of passive smoking were selected by the MeSH heading tobacco smoke pollution and/or relevant text words in the title, keywords or abstract. We then combined the results from the searches with the studies identified and included in the previous review [3].

### Inclusion and exclusion criteria

Two authors (AH & TM, or AH & JLB) independently reviewed the titles and abstracts identified from the searches, and identified all studies meeting the following inclusion criteria: (a) the design was a comparative epidemiological study (case-control, cross-sectional or cohort design); (b) LRI, pneumonia, bronchitis, bronchiolitis or acute respiratory infection, either by parental report or clinical diagnosis, was presented as an outcome; (c) passive smoke exposure was ascertained by self report and/or biochemical validation of parental smoking. We excluded studies that were not primary reports (such as systematic reviews and commentaries); or in which asthma, wheeze, proven infection with respiratory syncytial virus rather than clinically diagnosed bronchiolitis, or death from LRI were identified as the sole outcome; or in which the majority of infants in the study were over the age of two years. Following the title and abstract review, two of three researchers (LLJ, AH, and/or JLB) independently reviewed the full text, excluding irrelevant papers as appropriate. Disagreements were resolved through group discussion. Data relating to study design, methods, definition of LRI outcome, characteristics of reference group, ascertainment of passive smoke exposure, passive smoke source, and timing of exposure, location of study, and age of study population, were extracted using a previously piloted data extraction form and entered into a standardised database.

### Assessment of methodological quality

Studies that met the inclusion criteria were independently scored for methodological quality using the Cochrane Collaboration Non-Randomized Studies Working Group recognised Newcastle-Ottawa Quality Assessment Scale [6] by two reviewers. This scale is based on three broad categories relating to the selection of the study sample (four points); the comparability of the sample groups (two points); and the ascertainment of either the exposure (for case-control (three points) and cross-sectional studies (two points)) or the outcome (for cohort studies (three points). Thus, cross-sectional studies were rated out of a total of eight points and case-control and cohort studies out of a total of nine points. A score of seven or more was chosen *a priori *to indicate high methodological quality.

### Statistical analysis

Data were analyzed to yield effect estimates either using unadjusted (crude) odds ratios (OR) from extracted data from the publications, or where possible, adjusted ORs. Meta-analysis was carried out to estimate the effects on the risk of LRI of smoking by the mother only, father only, both parents, and any household member. Studies which clearly defined maternal smoking as pre- or post-natal were analysed separately. Random effects models [7] were used to calculate a pooled OR with 95% confidence intervals (CI) because the effect estimates were expected to be heterogeneous due to differences in the populations and exposures in the studies. Heterogeneity between study estimates was assessed using established methods (I^2^) [8]. To explore reasons for heterogeneity between the studies, sub-group analyses were used to assess the roles of disease outcome (LRI, pneumonia, bronchitis, bronchiolitis, or acute respiratory infection), study type (cohort, cross-sectional, or case-control), study publication date (pre versus post 1997), methodological quality (lower versus higher), and method of ascertainment of passive smoke exposure (self reported versus biochemical validation). Publication bias was assessed visually using a funnel plot for the association between exposure to household passive smoke and the risk of LRI. Data were analyzed using Review Manager, version 5.0.23 ((RevMan), Copenhagen, The Nordic Cochrane Centre, The Cochrane Collaboration). P values less than 0.05 were considered statistically significant. This analysis was performed in accordance with the Meta-Analysis of Observational Studies in Epidemiology (MOOSE) guidelines [9].

## Results

**Figure 1:**
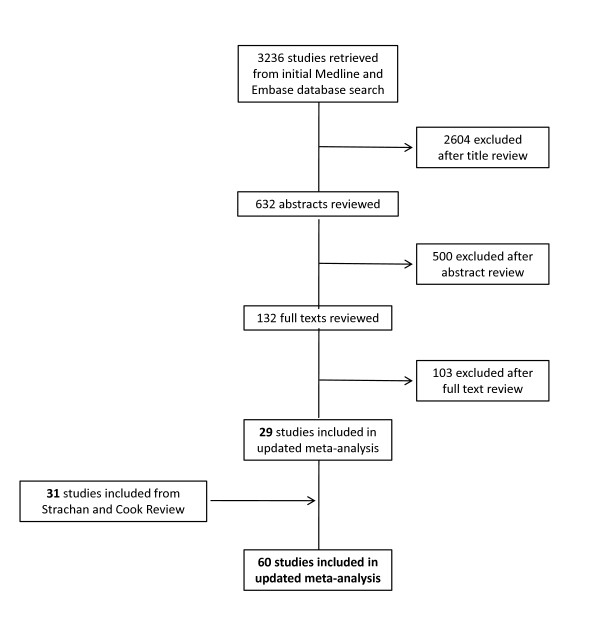
**Flow diagram of included and excluded studies**.

Our post 1997 literature searches identified an initial sample of 3236 papers, of which 132 were deemed eligible for further review on the basis of their title and abstract. One hundred and three of these studies were excluded after full text review because they were either: [a] not primary studies but editorials, letters or commentaries; [b] the majority of infants in the study sample were older than two years; [c] the definition of the outcome was not lower respiratory infection; or [d] there were insufficient or unusable data presented in the paper. We thus identified a total of 29 papers published between 1997 and the end of November 2010 which met our inclusion criteria of a comparative epidemiological study assessing passive smoke exposure and the risk of lower respiratory infection in infants less than two years of age. Of the 38 papers included in the original Strachan and Cook meta-analysis [3], seven did not meet our inclusion criteria, because wheeze was recorded as the primary outcome [10-15], or there were problems with recall bias [16]. We thus identified a total of 60 studies for inclusion in the present meta-analysis [see Additional file 1 and Figure 1].

Over half of the included studies [17-47] used data from cohorts, primarily birth cohorts; 15 studies [48-62] used a case-control design and 13 studies [63-75] were cross-sectional surveys. The LRI outcome reported was acute respiratory infection in seven studies [19,23,31,42,61,63,72], bronchiolitis in ten studies [36,48-50,53,55,56,59,64,73], bronchitis in ten studies [20,24,27,28,33,57,66,70,71,76], pneumonia in three [54,60,75], and in 30 studies the type of lower respiratory infection was not specified [17,18,21,22,25,26,29,30,32,34,35,37-41,43-47,51,52,58,62,65,67-69,74]. Studies measured infant exposure to passive smoke either by self-report [17-22,24-28,30-34,36,38-40,42,43,45,47-51,53-57,59-72,74-76], independent observation [23], or by biochemically validated measures of nicotine metabolites such as cotinine [35,37,41,44,46,52,58,73]. Thirty studies [17,18,24,25,29,34,35,38,40,43,46,48-50,52,53,56-62,66-70,75,76] adjusted for the infant's age in the analysis and 46 studies [17-22,24,26,28-35,37-39,43,45-50,52,56-71,73-75] adjusted for other potential confounding variables, such as breast feeding, maternal age, infant gender, allergy status, socio-economic status, and maternal education.

### Methodological quality of studies and publication bias

**Figure 2:**
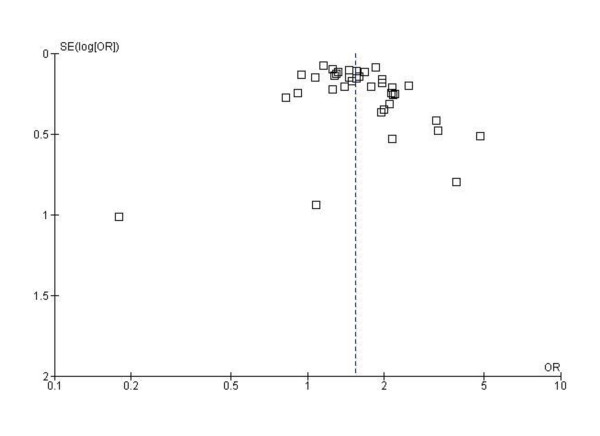
**Funnel plot for household passive smoke exposure against lower respiratory infection**. Plot shows the standard error of the odds ratio versus odds ratio for each study (random effects model). Vertical dotted lines indicate pooled effect estimate; and dots, individual studies.

The methodological quality of the 60 studies included in the meta-analysis, as judged by the Newcastle-Ottawa scale score, is presented in Additional file 1. The overall median score was six (range three to nine). Using the *a priori *chosen cut of seven to indicate high methodological quality, we judged 20 of the studies to be of high quality; and the remaining 40 to be of lower quality primarily due to a combination of a lack of biochemical validation of passive smoke exposure, lack of representativeness of the study sample, and/or lack of adjusted analyses. There was no evidence of publication bias identified from funnel plots. The funnel plot for any household exposure and the risk of LRI is presented in Figure 2.

### Effects of any household member smoking

**Figure 3:**
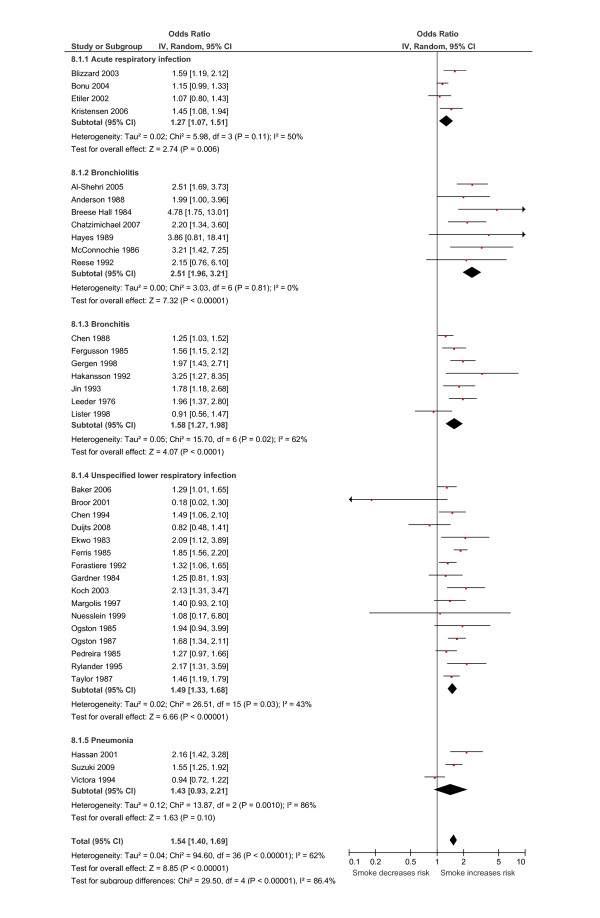
**Relationship between passive smoke exposure by any household member and the risk of lower respiratory infection (LRI) in infancy using a meta-analysis of comparative epidemiologic studies (Data are presented as odds ratios sub-grouped by the definition of LRI outcome)**. Squares denote the odds ratio (OR) for a single study with horizontal lines denoting 95% confidence intervals. The centre of the diamond denotes the pooled OR and the corners the 95% confidence intervals. An OR > 1 indicates a higher risk of the outcome in those exposed to passive smoke.

Exposure to smoking by any household member was associated with a statistically significant increase in the odds of LRI for infants under the age of two years, by 1.54 (95% CI 1.40 to 1.69; 37 studies; Figure 3). Moderate levels of heterogeneity (*I*^2^) were seen in the analysis (*I*^2 ^= 62%). Sub-analysis based on the definition of outcome showed that the increased risk of disease was predominantly due to a strong association between household passive smoke exposure and bronchiolitis (OR 2.51, 95% CI 1.96 to 3.21; 7 studies; Figure 3). Broadly similar, but lower increases in risk were estimated for all the other categories of LRI (ARI: OR 1.27, 95% CI 1.07 to 1.51; 4 studies; bronchitis: OR 1.58, 95% CI 1.27 to 1.98; 7 studies; ULRI: OR 1.49, 95% CI, 1.33 to 1.68; 16 studies; pneumonia: OR 1.43, 95% CI 0.93 to 2.21; 3 studies). All pooled odds ratios were significant except for pneumonia which was imprecisely estimated. Sub-group analysis based on study design showed similar pooled estimates of increased disease risk (cohort studies: OR 1.47, 95% CI 1.33 to 1.62; 17 studies; cross-sectional studies: OR 1.49, 95% CI 1.28 to 1.74; 11 studies; case-control studies: OR 2.01, 95% CI 1.31 to 3.10; 9 studies). Similar pooled estimates were also seen for sub-group analyses stratified by ascertainment of smoking status, date of publication and methodological quality.

### Effects of smoking by both parents

**Figure 4:**
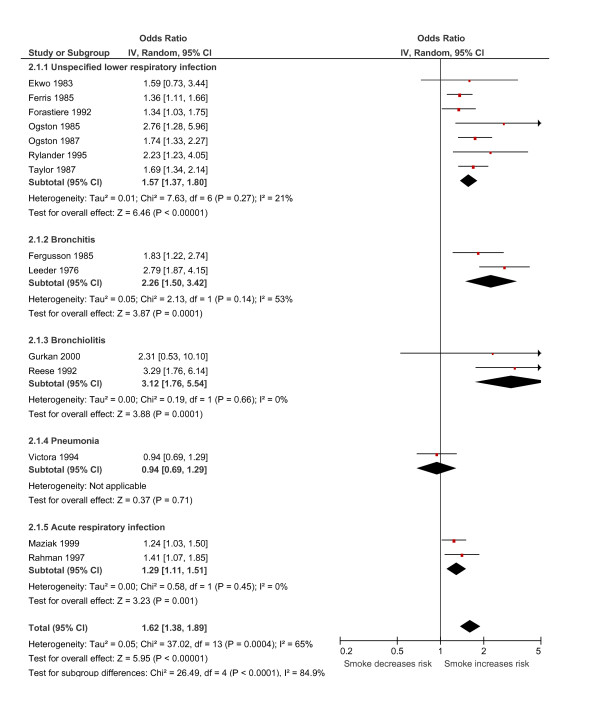
**Relationship between passive smoke exposure by both parents and the risk of lower respiratory infection (LRI) in infancy using a meta-analysis of comparative epidemiologic studies (Data are presented as odds ratios sub-grouped by the definition of LRI outcome)**. Squares denote the odds ratio (OR) for a single study with horizontal lines denoting 95% confidence intervals. The centre of the diamond denotes the pooled OR and the corners the 95% confidence intervals. An OR > 1 indicates a higher risk of the outcome in those exposed to passive smoke.

A pooled estimate of the 14 studies which defined exposure as both parents smoking demonstrated a statistically significant increase in the odds of LRI, by 1.62 (95% CI 1.38 to 1.89; Figure 4). Moderate levels of heterogeneity were seen between the studies (*I*^2 ^= 65%). Sub-group analysis based on the definition of outcome showed that the increased risk was again attributable in particular to a strong effect on the estimated risk of bronchiolitis (OR 3.12, 95% CI 1.76 to 5.54; 2 studies; Figure 4), and also bronchitis (OR 2.26, 95% CI 1.50 to 3.42; 2 studies; Figure 4). Pooled estimates for the other categories of LRI all identified statistically significant increases in risk (ARI: OR 1.29, 95% CI 1.11 to 1.51; 2 studies; ULRI: OR 1.57, 95% CI 1.37 to 1.80; 7 studies), again with the exception of pneumonia (p = 0.71, 1 study). In a sub-group analysis based on the method of ascertainment of passive smoke exposure, studies that used biochemically validated measures were significantly more likely (test for sub-group differences, p = 0.006) to show an increased risk of LRI (OR 2.69, 95% CI 1.75 to 4.13; 2 studies) than to studies that used self-reported data (OR 1.53, 95% CI 1.31 to 1.78; 12 studies). Similar pooled results were seen when the studies were categorised by methodological quality, date of publication, and by study design.

### Effects of paternal smoking

Meta-analysis of the 21 studies of paternal smoking demonstrated a statistically significant increase in the odds of LRI by 1.22 (95% CI 1.10 to 1.35). Pooled estimates for each of the outcome categories showed similar effect estimates by disease definition; however, these effects were significant only for bronchitis (OR 1.29, 95% CI 1.03 to 1.62; 3 studies) and unspecified lower respiratory infection (OR 1.26, 95% CI 1.08 to 1.45; 13 studies). In a sub-group analysis based on method of ascertainment of passive smoke exposure, similar pooled estimates for both self-reported (OR 1.24, 95% CI 1.13 to 1.36; 17 studies) and biologically validated (OR 1.26, 95% CI 0.62 to 2.54; 4 studies) measures were seen, although the latter was not statistically significant (p = 0.52). Similar pooled estimates were also shown for the sub-group analysis of methodological quality, study design and date of publication.

### Effects of pre-natal maternal smoking

Pooled estimates from the ten studies of pre-natal maternal smoking showed a statistically significant increase in the odds of LRI by 1.24 (95% CI 1.11 to 1.38). High levels of heterogeneity were seen between the studies (*I*^2 ^= 77%). This effect was stronger in the single study of bronchitis as outcome (OR 2.44, 95% CI 1.74 to 3.40); effects on ARI (OR 1.54, 95% CI 1.12 to 2.11; 1 study) and ULRI (OR 1.12, 95% CI 1.04 to 1.21; 8 studies) were weaker. In a sub-group analysis based on method of ascertainment of passive smoke exposure, studies that used self-reported data showed a statistically significant increase in disease risk (OR 1.25, 95% CI 1.11 to 1.40; 8 studies), in contrast to studies that used biochemical validation (OR 1.07, 95% CI 0.61 to 1.90; 2 studies). Similar pooled estimates were shown for the sub-group analysis of methodological quality, and study design. All of the studies included in this exposure group were published after 1997.

### Effects of maternal smoking after birth

**Figure 5:**
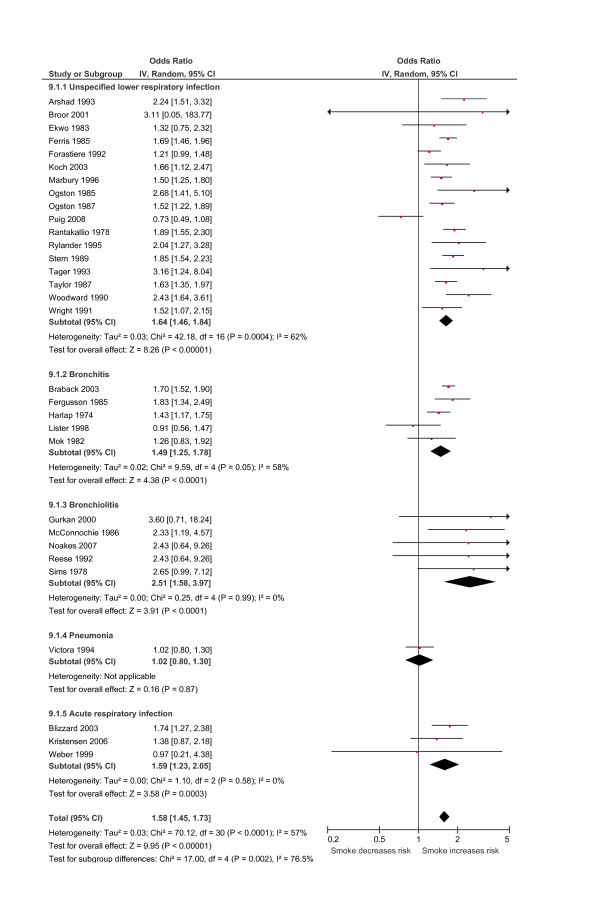
**Relationship between passive smoke exposure by maternal smoking after birth and the risk of lower respiratory infection (LRI) in infancy using a meta-analysis of comparative epidemiologic studies (Data are presented as odds ratios sub-grouped by the definition of LRI outcome)**. Squares denote the odds ratio (OR) for a single study with horizontal lines denoting 95% confidence intervals. The centre of the diamond denotes the pooled OR and the corners the 95% confidence intervals. An OR > 1 indicates a higher risk of the outcome in those exposed to passive smoke.

Maternal smoking after birth was associated with a statistically significant increase in odds of LRI, by 1.58 (95% CI 1.45 to 1.73; 31 studies; Figure 5). Sub-group analysis demonstrated a strong association between post-natal maternal smoking and bronchiolitis (OR 2.51, 95% CI 1.58 to 3.97; 5 studies; Figure 5). Pooled estimates for the other categories of LRI were similar and significant (ARI: OR 1.59, 95% CI 1.23 to 2.05; 3 studies; bronchitis: OR 1.49, 95% CI 1.25 to 1.78; 5 studies; ULRI: OR 1.64, 95% CI 1.46 to 1.84; 17 studies), with the exception of pneumonia (p = 0.87, 2 studies). Sub-group analysis based on study design showed similar pooled estimates of increased disease risk (cohort studies: OR 1.62, 95% CI 1.46 to 1.79; 16 studies; cross-sectional studies: OR 1.46, 95% CI 1.18 to 1.80; 6 studies; case-control studies: OR 1.73, 95% CI 1.23 to 2.44; 9 studies). In a sub-group analysis based on method of ascertainment of passive smoke exposure similar pooled estimates for both self-reported (OR 1.60, 95% CI 1.47 to 1.74; 26 studies) and biologically validated (OR 1.58, 95% CI 0.95 to 2.63; 5 studies) measures were seen, although the latter was not statistically significant. Similar pooled estimates were also shown for the sub-group analysis based on methodological quality and date of publication.

### Exposure-response relationships

An assessment of the relation between amount of exposure and disease risk was included in 26 of the 60 papers studied, quantifying exposure in terms of the numbers of cigarettes per day smoked by the source of exposure, the mean daily cigarette exposure of the infant, or by the number of smokers within the household. A positive, but not necessarily significant association was identified in 25 studies and an inverse relationship in one.

## Discussion

Passive smoking was recognised as a cause of lower respiratory infection in children in the US Surgeon General report of 2006 [1] and also in the UK Government SCOTH report [4]. Both reports drew on a series of systematic reviews and meta-analyses which for LRI originally included studies published up to 1997 [3], but was updated for the Surgeon General and SCOTH reports [1,4] by the inclusion of papers published to the end of 2001. The number of relevant studies has increased substantially since the original systematic review was published however, and the updated systematic review and meta-analysis described in the present study combines data from 31 of the studies used in the original review [3] with a further 29 studies published since 1997. This study demonstrates significant increases in the risk of LRI for smoking by the mother, father, both parents, and by any household member. These effects are typically strongest for bronchiolitis, and particularly in relation to maternal smoking. Pre-natal maternal smoking, which would be expected to be confounded with post-natal smoking because the majority of mothers who smoke through pregnancy continue to smoke post-delivery, also had an effect on LRI risk but this was weaker than most post-natal effect estimates. This indicates that post-natal tobacco smoke exposure, rather than exposure to blood-borne tobacco toxins *in utero*, is more likely to be the underlying cause of lower respiratory infections such as bronchiolitis in infancy.

The larger number of studies now available allowed us to explore effects on individual diagnoses included in the LRI category, and we found that the effect of passive smoking was typically strongest for bronchiolitis, and in some cases bronchitis. The magnitudes of the effects we detected were broadly consistent with the original review [3] though slightly smaller for post-natal maternal smoking (1.58 versus 1.72) and paternal smoking (1.22 versus 1.29). This may indicate that publication bias could have increased the magnitude of these earlier estimates; however, our funnel plot analysis for passive smoke exposure by any household member indicated that publication bias is unlikely to have had a marked effect on the results of the present study.

Our findings are likely to be representative estimates of the true effects of passive smoking on the risk of LRI in infancy since they are based on results of a comprehensive search, including data identified through hand searching of reference lists and previous reviews. However, there are limitations to this review. We elected to keep methods consistent with the original strategy [3] and only included studies written in English in the meta-analyses. Additionally, we were inevitably limited in the range of confounding factors that could be adjusted for in our analyses. Although the high quality studies generally adjusted for maternal age and socioeconomic status; other potential confounders, such as smoking by other individuals in the household, were not consistently adjusted for in the analyses of the individual effects of paternal and maternal smoking.

## Conclusions

This study thus confirms that exposure to all types of passive smoke, in particular maternal smoking, causes a statistically significant increase in the risk of infants developing lower respiratory infections in the first two years of life, and provides further precision in the estimates of the magnitudes of those effects in relation to differences in the source and extent of passive smoking in the home. Importantly, the study also identifies clinically-diagnosed bronchiolitis as a particular consequence of exposure, and one which can cause significant morbidity and in some cases mortality. Lower respiratory infections are common in infants, resulting, for example, in over 33,000 hospital admissions in infants aged under two years in England alone, where about 10% are estimated to be due to passive smoke exposure [5]. These additional hospital admissions are a significant public health burden all of which are avoidable. It is thus clear that there is a need for renewed efforts to prevent the exposure of infants to passive smoke, both during and after pregnancy.

## Competing interests

The authors declare that they have no competing interests.

## Authors' contributions

LLJ reviewed the full text articles, extracted data and wrote the initial draft of the manuscript. AH conducted the literature search, reviewed titles, abstracts and full text articles and contributed to the extraction of data. TM reviewed titles and abstracts and provided critical revision of the manuscript. DGC and JB contributed to the critical revision of the manuscript. JLB reviewed titles, abstracts and full text articles, extracted data and conducted the statistical analysis and provided critical revision of the manuscript. All authors read and approved the final manuscript.

## Supplementary Material

Additional file 1 **Summary of studies included in the meta-analysis**. The data provided represent a summary of each of the studies included in the updated meta-analysis. Click here for file
